# The Role of Diffusion Tensor MR Imaging (DTI) of the Brain in Diagnosing Autism Spectrum Disorder: Promising Results

**DOI:** 10.3390/s21248171

**Published:** 2021-12-07

**Authors:** Yaser ElNakieb, Mohamed T. Ali, Ahmed Elnakib, Ahmed Shalaby, Ahmed Soliman, Ali Mahmoud, Mohammed Ghazal, Gregory Neal Barnes, Ayman El-Baz

**Affiliations:** 1 Bioengineering Department, University of Louisville, Louisville, KY 40292, USA; y.elnakieb@louisville.edu (Y.E.); mtali003@louisville.edu (M.T.A.); aaelna02@louisville.edu (A.E.); ahmed.shalaby@louisville.edu (A.S.); ahmed.soliman@louisville.edu (A.S.); ahmahm01@louisville.edu (A.M.); 2 Department of Electrical and Computer Engineering, Abu Dhabi University, Abu Dhabi 59911, United Arab Emirates; mohammed.ghazal@adu.ac.ae; 3 Department of Neurology Pediatric Research Institute, University of Louisville, Louisville, KY 40202, USA; gregory.barnes@louisville.edu

**Keywords:** autism spectrum disorder (ASD), DTI, neuroimaging, ABIDE-II, diagnosis

## Abstract

Autism spectrum disorder (ASD) is a combination of developmental anomalies that causes social and behavioral impairments, affecting around 2% of US children. Common symptoms include difficulties in communications, interactions, and behavioral disabilities. The onset of symptoms can start in early childhood, yet repeated visits to a pediatric specialist are needed before reaching a diagnosis. Still, this diagnosis is usually subjective, and scores can vary from one specialist to another. Previous literature suggests differences in brain development, environmental, and/or genetic factors play a role in developing autism, yet scientists still do not know exactly the pathology of this disorder. Currently, the gold standard diagnosis of ASD is a set of diagnostic evaluations, such as the Autism Diagnostic Observation Schedule (ADOS) or Autism Diagnostic Interview–Revised (ADI-R) report. These gold standard diagnostic instruments are an intensive, lengthy, and subjective process that involves a set of behavioral and communications tests and clinical history information conducted by a team of qualified clinicians. Emerging advancements in neuroimaging and machine learning techniques can provide a fast and objective alternative to conventional repetitive observational assessments. This paper provides a thorough study of implementing feature engineering tools to find discriminant insights from brain imaging of white matter connectivity and using a machine learning framework for an accurate classification of autistic individuals. This work highlights important findings of impacted brain areas that contribute to an autism diagnosis and presents promising accuracy results. We verified our proposed framework on a large publicly available DTI dataset of 225 subjects from the Autism Brain Imaging Data Exchange-II (ABIDE-II) initiative, achieving a high global balanced accuracy over the 5 sites of up to 99% with 5-fold cross validation. The data used was slightly unbalanced, including 125 autistic subjects and 100 typically developed (TD) ones. The achieved balanced accuracy of the proposed technique is the highest in the literature, which elucidates the importance of feature engineering steps involved in extracting useful knowledge and the promising potentials of adopting neuroimaging for the diagnosis of autism.

## 1. Introduction

Autism spectrum disorder (ASD), famously known as just autism, is a pervasive developmental disorder manifested as problems in social interactions and communications, both verbal and non-verbal [1,2,3]. While there are no fully known causes of autism etiology, many hypotheses and theories exist. Regardless of the minutiae, it is believed that autism is a complex interaction between different genetic and environmental factors [4]. Current approved diagnosis techniques require significant clinical experience, assessing different aspects via a standard testing/scoring system, such as the ADOS [5] or ADI-R [6]. Those tests are subjective and can be time consuming and challenging, with limited accuracy of around 80–85% [7]. Furthermore, clinicians may not always agree with the results of those tests [8]. This is our main motivation for developing a neuroimaging-based alternative that can provide a non-subjective evaluation that may help clinicians reach a faster, more reliable diagnosis. Previous neurobiological studies investigated connections between ASD and underlying structure, trying to describe brain abnormalities associated with autism traits. Since the emergence of MRI, plenty of studies appeared to investigate connections between ASD and underlying brain features, either shape and volume features using structural MRI [9], or white matter (WM) diffusivity [10] anomalies using DTI, while others performed correlations of ASD with either task-based or resting-state functionality [11] using functional MRI (fMRI). In this paper, we will introduce our DTI-based algorithm for assessing ASD with the help of the ABIDE-II dataset.

DTI has been gaining rising popularity through the past couple of decades, especially for brain related disorders, as it provides a non-invasive way of characterizing the connective tracts inside the brain between different areas. It quantifies the diffusion patterns inside the white matter (WM). White matter mainly consists of axons of neurons (nerve fibers), and with the human brain containing hundreds of billions of neurons, the structure of WM is truly complex. The WM represents the axonal fibers carrying neural signals between various brain regions and between the brain and spinal cord through the brainstem. The organization of such a complex network contains a wealth of information; still, the current resolution for conventional MRI technologies cannot capture such small details, which are typically less than a micrometer to only few micrometers. Nevertheless, DTI provides diffusion measures that gives information about the tractography of the brain.

DTI’s most used parameters [12] include fractional anisotropy (FA), mean diffusivity (MD), and sometimes also “radial” and “axial” diffusivities. These parameters actually describe the diffusion of water inside the brain, and since water diffusion is restricted outside of fiber tracts, this translates into indirect information regarding the micro-structure and connectivity of WM [13]. Additionally, some derived features are also used to characterize other diffusion measures in WM tracts, such as tensor trace, skewness, rotational invariance, and many others [14]. Abounding previous literature has noted WM abnormalities associated with autism, often as differences in WM micro-architecture across some local brain areas. For instance, differences in FA values were reported by Wolff et al. [15] between ASD and typically developed (TD) infants. Using DTI, Barnea et al. [11] compared WM structure of ASD to normal TD, accounting for IQ, age and gender. They reported reduced FA in areas affiliated with social cognition in ASD, but found no difference for MD values. The role of MD values was identified by Alexander et al. [10], as they reported reduced FA values backed by an overall increase in MD across the corpus callosum for ASD vs. non-ASD individuals. Lee et al. [16] also reported higher MD values accompanied with reduced FA in autistic subjects, as well as higher radial diffusivity. In [17], a sample of 38 infants from the Infant Brain Imaging Study (IBIS) were used for the diagnosis of autism using spherical harmonics. Another study of ASD children [18] found, again, significantly lower FA in ASD subjects and correspondingly greater MD in frontal lobe WM. A separate study of 45 autistic subjects and 30 TDs manifested diagnostic potential when the authors split ASD to language impaired and non-language impaired groups based on FA and MD, achieving an accuracy of up to 80% [19].

Aside from classical analysis studies, plenty of studies have employed ML techniques for ASD classification. The whole ABIDE-I f-MRI dataset was tested with a refined deep learning model that was introduced by Heinsfeld et al. [20] that exceeded the previous state-of-the-art performance, achieving 70% accuracy. Khosla [21] presented another deep learning algorithm using a volumetric convolutional neural network that fits non-linear predictive models on 3D resting state fMRI (rs-fMRI) input and recorded a classification accuracy of up to 73% on ABIDE-I rs-fMRI data. In [22], the authors proposed framework exploiting features from both structural MRI (sMRI) and fMRI applied on 185 subjects from the National Database for Autism Research (NDAR), achieving 81% accuracy fusing both modalities. While most of those works relied on sMRI and/or fMRI, the focus of our paper is using DTI. DTI micro-architectural features were incorporated in another large recent study on 263 NDAR subjects for the diagnosis of autism, achieving accuracy of up to 73% [23]. Up to now, most of the published work regarding autism classification used ABIDE-I, and very few studies used newer ABIDE-II data [21,24,25,26]. One study used one site of ABIDE-II only (San Diego State University cohort), and employed both fMRI and DTI imaging modalities using connectome features, accomplishing an accuracy of 72% [27]. We emphasize that the need to use more than one modality implicates added cost and scanning time. Another key contribution of this work is finding a best-fit dimensionality reduction technique. Having a very large feature space (*p*) with limited sample space, or subjects, in our case (*n*), is commonly known as the curse of dimensionality [28], which causes increased complexity of the models that easily results in overfitting, with less learning captured by the model. This phenomenon is very common with MRI imaging and medical data, where we have piles of data fields for a few number of patients, and sometimes is not handled correctly. The standard way to handle those data is by exploiting some sort of feature reduction algorithms  such as linear discriminant analysis (LDA) [29], principal component analysis (PCA) [30], or auto-encoders [20]. The common shortcoming is that they usually do not keep the interpretation of the original feature in the new feature space, making it hard to explain clinical connections for any classification decision, and thus, making it less attractive for a practical medical use. The feature reduction method needs to help clinicians make an informative decision and aid in understanding the pathological abnormalities of the brain of autistic subjects. Our work investigates the recursive feature elimination (RFE) technique, which recursively eliminates the least contributing features for classification, ending with a best subset. We extensively carried out plethora of experiments to reach a near-optimal configuration that led to the best classification, as validated on our dataset.

Despite the numerous studies of autism-related changes in white matter integrity, the objective of this work is to implement a comprehensive ML-CAD system that, besides its ability to classify ASD vs. TD subjects, identifies brain areas correlated with autism, and was validated on a big, publicly available dataset using DTI data. The proposed algorithm employed a thorough feature selection using recursive feature elimination with cross-validation (RFE-CV) using four different kernels (SVM with linear kernel (LSVM), random forest (RF), and logistic regression (LR), either with a $l_{1}$-norm (LR1), or LR with $l_{2}$-norm (LR2)), and performed hyper-parameter optimization on eight different classification techniques. The best candidate configurations were validated using random splits of different k-folds’ cross-validation to identify the global ML model alongside the global imaging bio-markers associated with ASD. Our main motivation behind this work is to present a reliable system that can help physicians better understand individuals with autism, allowing earlier and more personalized treatment plans. The rest of this paper is organized as follows: Section 2 presents the details of the pipeline of the proposed algorithm, while the experimental results are introduced in Section 3 for the ABIDE-II diffusion MRI data. Finally, Section 4 provides a discussion and the conclusions of the paper.

## 2. Methodology

**Figure 1:**
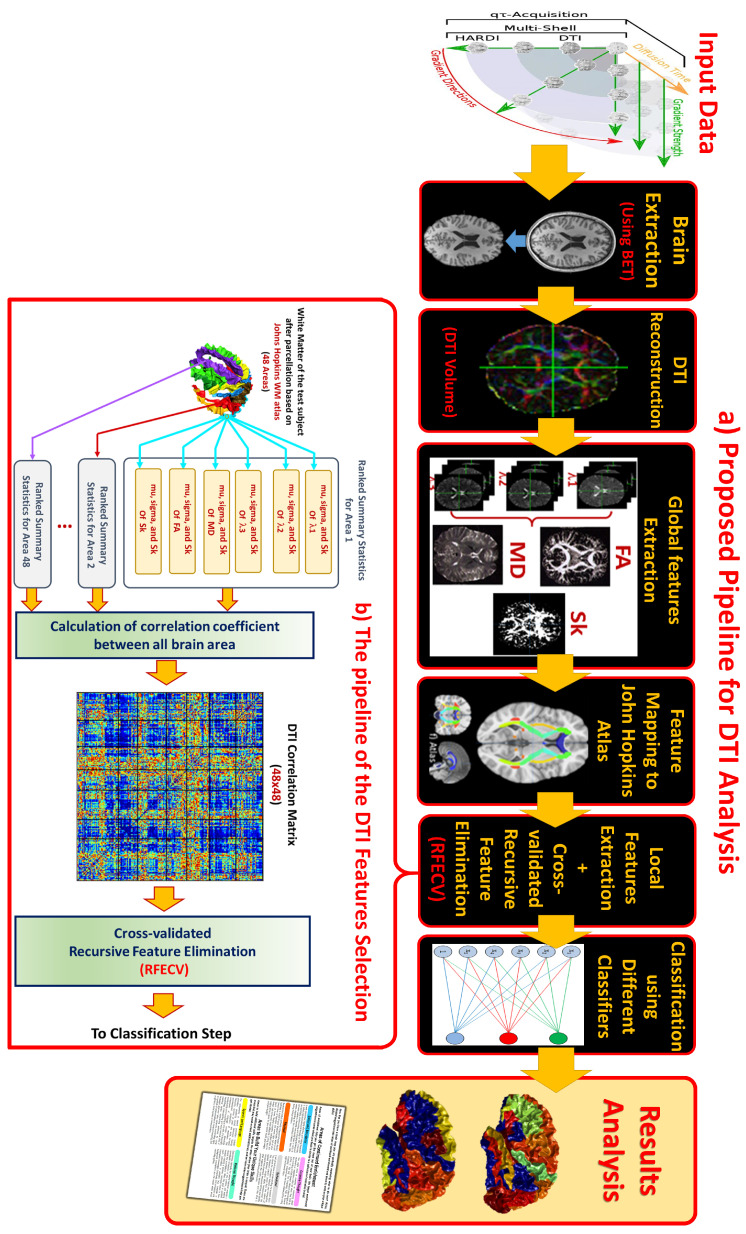
(**a**) Pipeline of the DTI-diagnosis algorithm. (**b**) Usage of the new derived feature representation $\hat{F}$ and feature selection before classification.

A visualization of the pipeline of the whole framework is presented in Figure 1. It starts with pre-processing of each subject’s input volumes, and is then followed by DTI parameter calculations, feature extraction and mapping to a WM atlas to get local features. This is followed by using two different feature representations, to be used in feature selection and classification steps. The following subsections provide details of these multi-stage processes until reaching a final diagnosis.

### 2.1. Data Used

This work utilized DTI data from the Autism Brain Imaging Data Exchange (ABIDE)-II dataset. ABIDE-II is a recent publicly available dataset that aggregates MRI data (sMRI, fMRI, and DTI) for autism studies across different multiple sites. ABIDE-II contains data from around 19 sites for more than 1000 subjects; half of them are autistic individuals. Working on a publicly available dataset facilitates replicating results and increases the reliability of our findings. ABIDE-II is considered a large dataset, which increases the power of our study. We selected datasets that involved DTI data, which included 6 datasets, namely: Barrow Neurological Institute (BNI), NYU Langone Medical Center 1 (NYU1), NYU Langone Medical Center sample 2 (NYU2), San Diego State University (SDSU), Institut Pasteur and Robert Debré Hospital (IP), and Trinity Centre for Health Sciences (TCD). IP DTI data bvals (diffusion gradient strength per volume values) and bvecs (diffusion gradient directions per volume values) were missing a value, so we excluded it, and used the remaining five sites. Those 5 sites originally had 284 subjects with DTI imaging data, and ended with 225 subjects of them after cleaning the data, on which we applied the steps of our pipeline, as we will elaborate on in the next subsections.

### 2.2. Pre-Processing

#### 2.2.1. Input Image Preparation

After deciding which sites to work on, we downloaded their available data, which came organized as folders labeled by subject ID containing imaging data. We located subjects that had DTI data, copying the relevant image nii files along with bvals and bvecs to the working directory to be pre-processed.

#### 2.2.2. Skull Stripping

The goal of the skull stripping step is to remove non-brain tissues (e.g., skull, scalp, dura, …) from the image volumes, extracting only the brain. This automated process was implemented using the brain extraction tool (BET) algorithm [31] from FSL tools, generating the binary masks and using default parameters with a fractional intensity threshold of 0.25.

#### 2.2.3. Eddy Current Correction

Eddy currents are induced currents due to gradient fields in the x, y, z directions that result in visible image artifacts that usually blur the boundaries between gray and white matter. Diffusion-weighted imaging is usually affected by this phenomenon, and an eddy current correction step is commonly implemented. For this purpose, we used the eddy current correction tool ‘eddy’ available through FSL [32] to correct for both common artifacts, including adjusting for induced currents and also for subject movement during the scan, across sections.

### 2.3. Feature Calculation

After having the diffusion-weighted volumes cleaned of non-brain tissues and common artifacts, we run DTI calculations to get the DTI diffusion tensor, its eigenvalues, and other metrics. For each voxel, diffusion can be represented by a 3 by 3 tensor, which describes the diffusion pattern at each point in 3D space. From this tensor, a more common metric, namely eigenvalues, is used to represent the magnitude of diffusion along 3 major perpendicular directions of its eigenvectors. The largest eigenvalue, $\lambda_{1}$, along with its eigenvector, $v_{1}$, represent the magnitude and direction of the primary direction of diffusion (along the fiber tract), while the other two represent radial diffusion perpendicular to the main one [33]. Other derived metrics, such as fractional anisotropy, mean diffusivity, skewness, and many others are commonly used to represent other characteristics of the diffusion. In our work, we included the following 6 metrics to describe our white matter micro-architecture:

1. Fractional anisotropy (FA): Measures the degree of anisotropy of the diffusion, with zero representing completely isotropic diffusion, and one representing a directional diffusion [33];
2. Mean diffusivity (MD): Average magnitude of diffusion at each point, independent of the direction. $\mathrm{MD}=\frac{1}{3}\sum_{i=1}^{3}\lambda_{i}$;
3. Axial diffusivity (AD): Magnitude of diffusion along the major axis; $\mathrm{AD}=\lambda_{1}$;
4. & 5. Radial diffusivities: Magnitude of diffusion along the two perpendicular axes to AD: RDs = [*λ*_2_, *λ*_3_];
6. Tensor skewness: A higher order moment of diffusion, revealing more information not captured by lower order ones. [14];
  $\mathrm{TSkew}=\frac{1}{3}\sum_{i=1}^{3}{\left(\lambda_{i}-MD\right)}^{3}$.

For the first five features, the dtifit tool, part of the FSL package, was used to calculate the diffusion tensors along with eigenvalues, eigenvectors, FA, and MD. Tensor skewness (Tskew) was calculated using Matlab 2021a, as it was not provided through the previous tool. At this point, each subject is represented by six volumes, each comprising hundreds of thousands of raw voxel values.

#### Data Cleaning

In the previous parts of the pipeline, some subjects failed during volume size validation, BET and DTI calculations, or regional feature extraction, either with an error in the prepossessing or yielding a non-complete brain, identified by having more zero values, or “blanks”, than it should. Excluding those subject from further processing, we ended up with 225 subjects that will be used for the rest of this work. Subject IDs along with age, label, IQ, and gender for all subjects used in this study are provided as a Supplemental Material, Table S3.

### 2.4. Atlas-Based Segmentation

Having each subject represented by its six volumes per voxel feature, now we need to assign those features to local brain areas. For this purpose, the white matter atlas ICBM-DTI-81, defined by Johns Hopkins University [34], is used. The JHU ICBM-DTI-81 WM atlas uses ICBM coordinates and defines 48 white matter areas. Those areas were originally hand-segmented from the average of diffusion MRI tensors of different 81 subjects. To locate local anatomical regions in each subject space, we implemented an atlas-based segmentation approach, where we preformed atlas registration for area localization. Registration from the atlas space to subject’s space was performed in two iterations: a rigid transformation then an affine transformation. The objective of the rigid registration in the first iteration is just to find an initial alignment, not changing the size or shape, that will be used for next step. Then, an affine transformation is found to improve upon the initial estimation by providing a higher degree of freedom for a more generic linear transformation that enables the object’s size and shape to be adjusted. This two-step registration task was implemented using DTI-TK software [35] using normalized mutual information measures with a 4 mm × 4 mm × 4 mm sampling distance and 1% tolerance. DTI-TK also enables interoperability with FSL software used in preprocessing. The found transformation was then applied to atlas labels, hence providing WM areas mask at each subject space. Those masks were used to define local features for those 48 areas. This segmentation technique provides a fast automated solution, enabling easy application to new subjects or datasets, with less error.

### 2.5. Feature Representation

At this point, each subject is represented by six features per 48 areas. Each of those features are per-voxel raw features, and their length, in tens of thousands, varies between areas. The first step is to convert those raw features into a better representation with the goal of reducing the number while keeping the most important aspects capturing underlying information. For this purpose, we replaced per-voxel features of each area with three summary statistics of underlying distribution, namely, the mean (μ), standard deviation (σ), and skewness (*sk*), where μ aims to the capture central tendency, σ captures the dispersion of values around this mean, and sk aims to measure the asymmetry of the data around this mean. At the end of this step, our feature matrix *F*, for each subject *i*, can be represented as a 48 by 18 matrix, as follows:

$$F_{i}=\left[\begin{array}{ccccc} \mu_{FA_{1}} & \sigma_{FA_{1}} & sk_{FA_{1}} & \ldots & sk_{Tskew_{1}}\\ \mu_{FA_{2}} & \sigma_{FA_{2}} & sk_{FA_{2}} & \ldots & sk_{Tskew_{2}}\\ \ldots & \ldots & \ldots & \ddots & \ldots \\ \mu_{FA_{48}} & \sigma_{FA_{48}} & sk_{FA_{48}} & \ldots & sk_{Tskew_{48}} \end{array}\right]$$

where $F_{i}$ is the feature matrix for subject *i* using the first feature representation described above. Each element in this matrix is a summary statistic (baseline: μ/σ/sk) for one of the six features (subscript: FA/MD/Tskew) for an area from 1 to 48 (sub-subscript index).

#### 2.5.1. Feature Engineering

Instead of directly using per-area summary statistics features, we developed an enhanced representation that captures latent relative relationships between brain areas. We calculated Pearson correlation coefficient between each pair of brain areas l,m, and use this correlation matrix as our feature matrix. Therefore, for each subject *i*, $\rho_{l,m}=corr\left(F_{i}\left(l,:\right),F_{i}\left(m,:\right)\right)$. Although this step increased the number of features per subject slightly [from 48 × 18 = 864 to (48 × 47/2) = 1128], it helped in boosting the performance of the classification, as we will see in the results. This novel representation, using interactions, is considered a key contribution that helped in improving the performance. The new second feature matrix $F_{2\_i}$ for subject *i* is now represented by:

$$F_{2\_i}=\left[\begin{array}{cccc} \rho_{1,1} & \rho_{1,2} & \ldots & \rho_{1,48}\\ \rho_{2,1} & \rho_{2,2} & \ldots & \rho_{2,48}\\ \vdots & \vdots & \ddots & \vdots \\ \rho_{48,1} & \rho_{48,2} & \ldots & \rho_{48,48} \end{array}\right]$$

where each element in this matrix $\rho_{i,j}$ is a correlation between the summary statistics vectors of the two areas i,j. We highlight that only the upper triangle (*U*) of this new feature matrix (or lower *L*, because of symmetry) is used in subsequent steps, as the rest is redundant because of symmetry. Serializing those 1128 features, we can represent the final feature matrix for all 225 subjects as $\hat{F}$ with size 225×1128, where each row is the concatenated calculated correlations for one subject. Figure 1b illustrates those steps. In addition to the data matrix, we have another column vector *y* denoting the labels of each subject, whether ASD ($y_{i}$ = 1) or TD ($y_{i}$ = 0).

$$y=\left[\begin{array}{ccccc} y_{1}, & y_{2}, & y_{3}, & \ldots , & y_{225} \end{array}\right]\;$$

#### 2.5.2. Feature Reduction: RFE-CV

The feature space (1128 correlations) is quite large relative to our sample size (225 subjects). As we discussed earlier, the number of features relative to the number of subjects needs to be reduced, keeping the most informative features. While many feature reduction techniques, such as linear discriminant analysis, principal component analysis, or autoencoders, can perform this task, they transform the feature space into a new one that does not preserve the meanings of the original features. Building classification systems based on those new ambiguous features would sophisticate the ability to understand any clinical reasoning of classification results, hence making it less beneficial and reasonable to physicians in generating an informative decision or understanding the underlying pathological abnormalities of an autistic brain. We employed the recursive feature elimination (RFE) technique, where only a subset of features is selected. RFE is a feature selection algorithm based on feature ranking with recursive feature elimination. The principle behind RFE is fitting a classification model, ranking the features by the model’s scoring, then eliminating the weakest features recursively to find the optimal number of features to be selected. Cross validation is used with RFE (RFE-CV), where data is split into k-folds, features are scored based on different data subsets, and then the best scoring across the k-folds is selected. The target optimization scoring metric (whether accuracy, balanced accuracy, *f*1, weighted *f*1, precision, recall, …) can be specified, and here, we used balanced accuracy with *k* = 10 folds for optimization. The algorithm then finds the optimal *n* significant features to be selected that maximizes the average classification performance according to the target metric [36,37]. To find the best architecture of RFE-CV that best fits our problem, we tested four types of RFE-CV classifiers as kernels, namely linear SVM (LSVM), random forest (RF), logistic regression (LR) with $l_{1}$-norm (LR1), and LR with $l_{2}$-norm (LR2), on the two feature representations we have (original summary statistics $F_{i}$ of 225×864 and correlations $F_{2\_i}$ of 225×1128). Thus, we obtained estimates using four different models, each selecting features according to its classifier independently, and providing average cross-validated scores for 10-folds; then we evaluated the performance of eight models to select which model to use for further processing.

### 2.6. Classification

After having *n* selected features for each of 8 models representing the top prominent features for distinguishing autistic brains, we set up a system of machine learning classifiers. We tested eight different classifier types, and performed hyper-parameter optimization for each one to end up with best parameter classifier model in terms of accuracy. We included both linear and non-linear classifiers to test both types of relationships between the two classes. The set of used classifiers are: (1) linear SVM (LSVM), (2) logistic regression (LR), (3) passive aggressive classifier (PAGG), (4) SVM with radial-basis kernel (RBF-SVM), (5) Gaussian naive Bayes (GNB), (6) random forest (RF), (7) XGboost (XGB), and (8) neural networks (NN). Classifiers 1–3 are linear classifiers, while the rest are non-linear. Classifiers 6 and 7 are ensemble-based classifiers, and for NN we included both shallow and deep configurations in our hyper-parameter search. For hyper-parameter optimization, after we selected only *n* features according to the previous RFE-CV step, we tested a set of different parameters with different ranges for each classifier. For this purpose, the input data is split into five folds to determine the best performance according to the average across those five folds. Therefore, for each classifier, using the selected features only, the following steps were performed: (i) split data into five folds, use four for training and one for testing each time, and for each parameters configuration, store the performance of the classifier for each fold; (ii) The balanced accuracy scoring is used to decide the best configuration; (iii) The best performing classifier is selected, and the hyper parameters along with its maximum average cross-validated score, and also standard deviation over folds, are highlighted. Table 1 shows the set of used hyper-parameters in the search associated with each classifier and their ranges. Algorithm 1 illustrates a step-by-step guide of the full implemented algorithm, and  Figure 1 summarizes a graphical illustration of the pipeline of the entire system.
**Algorithm 1** Diffusion tensor autism diagnosis algorithm.  1:∀ **subject’s data files: (NII+bval+bvec)**:  2:     1. Check for errors, check bval and bvec files.  3:     2. run pre-processing modules:  4:           (i) Run skull stripping using brain extraction tool (BET).  5:           (ii) Run FSL’s eddy current correction tool.  6:           (iii) Register the DTI IIT Human Brain Atlas to each subject space using DTI-TK tool, save transformations.  7:           (iv) Recheck for any generated errors or deformations.  8:     3. Feature Calculations:  9:           (i) Use FSL to calculate DTI tensor, scale units, calculate RDs, AD, FA, MD, Tskew volumes.10:           (ii) Apply resulted transformation on the JHU atlas labels to generate masks.11:           (iv) Use registered masks to extract each feature for each WM region.12:           (v) Calculate summary statistics (μ, σ, Sk) for each area for each feature ($\lambda_{1}$,$\lambda_{2}$,$\lambda_{3}$,FA,MD,Tskew), rank feature values across the different 48 brain areas, get a concatenated feature vector (3*6). Create feature matrix *F* to be used as a first variant of the input data matrix *X*.13:           (vi) Calculate correlations between feature vectors of each two areas to create feature matrix $F_{2}$.14:           (vii) From $F_{2}$: remove redundant correlations (*L* and diagonal) and concatenate *U* to create $\hat{F}$ to be used as a second variant of the input data matrix *X*.15:4. RFECV feature selection: for each feature representation, and for each RFE-CV kernel:16:       (i) Split input data X, labels y into k folds. Each time use one fold as $X_{test}$, $y_{test}$, rest as $X_{train}$, $y_{train}$.17:       (ii) Train the classifier using each $X_{train}$, $y_{train}$.18:       (iii) Get the balanced accuracy score of the trained classifier using $X_{test}$, $y_{test}$.19:       (iv) Calculate the cross-validated score and sort features based on importance.20:       (v) Remove the least important features from *X* matrices, and repeat the steps from (i) to (v) until only one feature exists.21:       (vi) Determine the *n* features that provided the best cross-validated score along with its hyper-parameters to be used for each of the kernels.22:5. Classification:23:       ∀ classifier, for each configuration of hyper-parameters:24:       (i) Split reduced $X_{select}$, with *n* selected features, into k folds, along with *y*.25:       (ii) Calculate the cross-validated score for each hyper-parameter’s configuration.26:       (iii) Determine best hyper-parameter configuration in terms of score for each classifier.27:       (iii) Find the best classifier/parameters, along with its used *n* features.

## 3. Results

As discussed in the data subsection, the ABIDE-II dataset [38] was used for the testing and validation of the above-mentioned methodology. ABIDE-II [38] provides hundreds of subjects’ brain imaging data (structural MRI, functional MRI, and DTI) to enhance research in autism spectrum disorder (ASD). DTI data used are only from the following five sites: IP, NYU1, NYU2, TCD, and SDSU. Diffusion-weighted MRI (dwMRI) scans for a total of 225 subjects were used: 125 ASDs and 100 TDs, with age ranges between 5.128 years and 46.6 years.

The four types of RFE-CV kernels (LSVM, LR1, LR2, and RF) were used to select features from the two different representations (summary statistics *F*, and correlations $\hat{F}$), and those features were used to train and test eight types of classifiers (LSVM, LR, PAGG, RBFSVM, GNB, RF, XGB, and NN). The hyper-parameter optimization step was carried out for each combination of [feature-RFECV kernel-classifier], using a grid search over the list of hyper-parameters on Table 1 with five-fold cross validation with the help of the GridSearchCV scikit learn toolkit. The aim of this search was to identify the best RFE-CV kernel in terms of accuracy, to be used for the final classification/validation stage. Based on the results of those 64 sets of combinations, we identified which setting best suits our data, then we investigated it with more validations, changing the splits and varying the number of folds.

**Figure 2:**
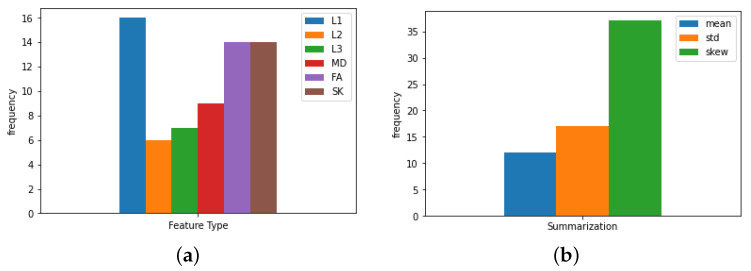
Histogram of types of selected summary statistic features. (**a**) for the occurances of each feature type, (**b**) for summary statistics occurrences.

Tables S1 and S2 in the Supplementary Materials show the full details of this round of experiments for both feature representations: summary statistics *F* and correlations $\hat{F}$, respectively. We notice that both LR1 and LR2 kernels almost failed to provide representative features in terms of accuracy results (accuracy ~60%). While the RF kernel provided us with moderate results (mostly above 70%), LSVM was the one we were searching for, achieving accuracies of up to 99% with $\hat{F}$ features. More importantly, we highlight that using our novel feature representation $\hat{F}$, we were able to achieve this high boost in classification results. To show which types of features were more representative, we show the histogram of the occurrence of each type of summary statistics appearing in selected features from *F* with LSVM RFE-CV used in Figure 2. The figure illustrates the efficacy of adding SK feature which appeared as important as the common FA metric, and points out coice of skewness as a relevant summary statistic.

Following these results, we will only use the LSVM RFE-CV kernel with $\hat{F}$ representation (correlations) for further investigations, as it shows better performance. We will fix the hyper-parameters of the eight classifiers to the ones we previously found on the first set of experiments (Table 2), and randomly re-split different settings of k-fold cross validation, with k = [2, 4, 5, 10], to test whether the achieved performance is highly dependent on the split and/or the subjects of previous experiment and see the effect of changing the proportion of train/test on the results.

Table 3 shows the final diagnostic accuracies of our proposed framework using our novel feature representation with the help of RFE-CV with the LSVM kernel, and Table 4 shows the area under the curve for each of the classifiers across different k-folds. Without a new optimization, using the same settings, and on new sets of random splits, our innovative algorithm was still able to provide up to 99% accuracy, which clearly manifested the strength of the presented algorithm.

**Figure 3:**
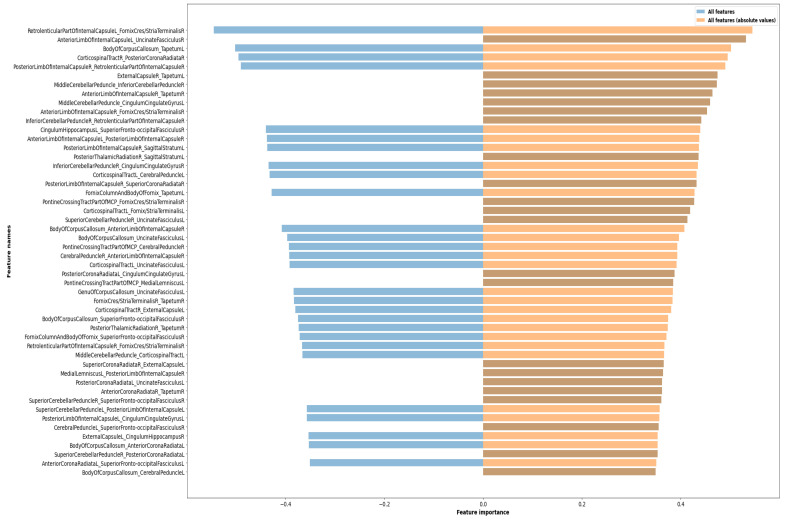
Sorted coefficient of importance for the top 50 selected features of the area pairs correlations.

Figure 3 illustrates the importance of the top selected features by our RFE-CV LSVM kernel. The bars in blue on the left indicate high negative correlation importance with our positive class (autism), while the ones in dark orange on the right indicates a positive importance coefficient. The longer the bars, the higher the coefficient, indicating more importance for features of this brain-area pair. Table 5 lists the name of the top twelve feature-pairs as ranked by our selection algorithm for easier identification. We can see that most of those brain areas already appear in the literature as correlating with the ASD phenotype. We already see some areas appear more than once in the top 12 pairs; we will discuss the importance of the highlighted brain areas more in the following section, Discussion.

## 4. Discussion and Conclusions

The proposed technique adopted in this study introduced a novel feature representation applied to a large number of subjects obtained from a publicly available dataset. We performed extensive experimentation to validate the results introduced through this paper, as well as paved the path for developing new frameworks that may benefit from our novel algorithm. In addition to the achieved promising results, in terms of high cross-validated balanced accuracy, we introduced the notion of interaction between brain areas’ micro-connectivity and its viability of reaching a better classification of autism. More importantly, we identified the brain-area pairs that mostly contributed to reaching the final decision. We highlight that those identified brain areas in Table 5 align with the corpus of findings from previous literature studying autism impairments. The uncinate fasciculus (uc) is a fiber pathway through the external capsule (ec) which links the ventral frontal cortex, in particular Brodmann areas 11 and 47, with the temporal pole, and differences in it were revealed in [39,40]. On the other hand, the middle cerebellar peduncle (mcp) carries signals from the cerebral cortex and subcortical regions, via the pontine nuclei, into the cerebellar cortex. The internal capsule (ic) microstructure was found to undergo an atypical developmental trajectory in autistic patients, manifested as increased connectivity from childhood to adulthood [41]. All parts cited in this study of the ic are involved in autism [41,42,43,44,45], and DTI changes have been correlated with autistic behaviors, including inattention, self injury, repetitive behaviors, and social deficits. In general, all white matter tracts identified here (Table 5, Figure 3) connect cortical (sensory motor cortex, frontal/occipital lobes, cingulate) and subcortical regions (thalamus, hippocampus, cerebellum), thereby contributing to deficits (inattention, self injury, repetitive behaviors, motor, social, memory, emotional regulation, and sensory impairments) found in autistic individuals [41,42,43]. Shukla et al. [45] identified reduced FA and increased RD in the ic and corpus callosum (cc) in children with autism. They also spotted increased MD in anterior and posterior limbs of ic. Significant differences in the AD of the stria terminalis (st) was reported by Yamagata et al. [46] between ASD and TD individuals. Reduced FA and increased RD of st was also reported in [40], and higher AD of st in TD children was noted in [43]. Differences in middle, inferior, and superior cerebellar peduncles [45,47,48,49] and the corpus callosum [43,45,47,50] were also reported in those previous studies.

The tapetum WM is part of the splenium fibers around the cc, providing connectivity between the temporal lobe, and was found to play a role in different mental disorders [51]. Reduced FA, increased RD, and decreased AD of the tapetum has been reported in ASD. Abnormalities in the corticospinal tract, corona radiata, external capsule, cingulum cingulate cyrus, cingulum hippocampus, and superior fronto-occipital fasciculus were noted in previous studies [13,23,40,42,44,49,52,53,54,55,56]. We stress that our findings are for brain regions’ interactions with others, following the idea of disrupted connectivity introduced by Vasa et al., and work normally when done in functional MRI experiments. In [57], Vasa et al. reviewed some of the current structural and functional connectivity ASD data to examine the “disrupted connectivity” theory. They identified and highlighted many confounding factors in the literature that could have affected this conclusion.

In conclusion, the classification framework presented accomplishes many objectives. It provides a high state of the art balanced accuracy on a public dataset, and interpretability, not only in providing a ASD/TD diagnosis, but also in identifying what areas contributes to such a classification. Those spotted brain areas can be reported early with the framework’s diagnosis to the physician, who can now make better informed decisions. We believe that this is an important aspect that would lead to a better understanding of the brain abnormalities associated with autism. The system we present is also scalable: adding more subjects that can be preprocessed and feature calculated independently, and fusion of an extra modality, such as structural MRI features or resting state functional MRI for the same subject, can be easily integrated. On the other hand, we stress that the robust results were obtained and validated using only five ABIDE-II sites, and adding more datasets should guarantee generalizability of our proposed framework, which can be a good direction for future work. Moreover, more sophisticated medical interpretation is needed not only to map those affected brain areas to TD vs. ASD, but also to correlate them with ADOS or similar scores, allowing more distinction per scored module. This may need integration with other imaging modalities such as sMRI or fMRI to incorporate different aspects (shape and functionality) to our classification framework, progressing towards an integrated system for autism assessment and providing better interpretation and understanding of underlying personalized diagnosis.

## Figures and Tables

**Table 1:** Used hyper-parameter values in a cross-validated grid search. Names between parentheses are parameter names in the ML package.

Classifier	Hyper-Parameter	Range/ Values
(1) LSVM	Regularization (C)	0.1, 1, 5, 10
	Loss function (loss)	L1, L2
	Penalization strategy (penalty)	squared_hinge, hinge
(2) LR	Penalization strategy (penalty)	L1, L2 elastic
	Regularization (C)	0.1, 1, 5, 10
	Solver algorithm (solver)	newton-cg, lbfgs, liblinear, sag, saga
(3) PassiveAgressive	Regularization (C)	0.1, 1, 5, 10
	N idle iteration before stop (n_iter_no_change)	1, 5, 10
(4) Nonlinear-SVM	Regularization (C)	0.1, 1, 5, 10
	Kernel used (kernel)	rbf, poly, sigmoid
	Polynomial kernel degree (degree)	2–6
	Kernel coefficient (gamma)	scale, auto
	Independent term in kernel function (coef0)	0.0, 0.01, 0.1, 1, 5, 10, 50, 100
(5) GNB	Default parameters	priors = None, var_smoothing = $1\times 10^{-9}$
(6) RF	Number of features to consider when looking for the best split (max_features)	auto, sqrt, log2
	Number of trees in the forest (n_estimators)	50, 100, 200, 500, 1000
	Function to measure the quality of a split (criterion)	gini, entropy
	Bootstrap samples when building trees (bootstrap)	True, False
	Min # of samples required to split an internal node (min_samples)	1, 2, 5, 10
(7) XGB	Which booster to use (booster)	gbtree, gblinear, dart
	Learning rate (learning_rate)	0.001, 0.01, 0.1, 0.3, 0.5, 1
	Min loss reduction required to make a further partition on a leaf node (gamma)	0, 0.1, 0.5, 1, 1.5, 2, 5, 20, 50, 100
	Min sum of instance weight needed in a child (min_child_weight)	0.1,0.5, 1, 5, 10
	Subsample ratio of columns when constructing each tree (colsample_bytree)	0.6, 0.8, 1.0
	L2 regularization term on weights (lambda)	0, 0.001, 0.5, 1, 10
	L1 regularization term on weights (alpha)	0, 0.001, 0.5, 1, 10
(8) NN	Hidden layer sizes (hidden_layer_sizes)	(150,100,50,), (100,50,25,), (100,)
	Activation function (activation)	tanh, relu, logistic
	Solver used for weight optimization (solver)	lbfgs, sgd, adam
	L2 regularization penalty (alpha)	0.0001,0.001,0.01, 0.05, 0.1, 0.5
	Initial learning rate (learning_rate)	constant, adaptive
	Exponential decay rate for estimates of first moment vector in adam (beta_1)	0, 0.001, 0.01, 0.1, 0.3, 0.5, 0.9
	Exponential decay rate for estimates of second moment vector in adam (beta_2)	0, 0.001, 0.01, 0.1, 0.3, 0.5, 0.9

**Table 2:** The fixed hyper-parameters found to optimize performance on the set of tested classifiers.

lSVM	{‘penalty’: ‘l2’, ‘loss’: ‘hinge’, ‘C’: 1}
pagg	{‘n_iter_no_change’: 5, ‘C’: 0.1}
LR	{‘solver’: ‘newton-cg’, ‘penalty’: ‘none’, ‘C’: 0.1}
XGB	{‘reg_lambda’: 0.001, ‘reg_alpha’: 0, ‘min_child_weight’: 10, ‘learning_rate’: 1, ‘gamma’: 0.1, ‘colsample_bytree’: 0.6, ‘booster’: ‘gblinear’}
GNB	defaults
SVC	{‘kernel’: ‘poly’, ‘gamma’: ‘scale’, ‘degree’: 3, ‘coef0’: 5, ‘C’: 0.1}
Rf	{n_estimators’: 50, ‘min_samples_split’: 2, ‘min_samples_leaf’: 0.1, ‘max_features’: ‘sqrt’, ‘criterion’: ‘entropy’, ‘bootstrap’: False}
nn	{‘solver’: ‘adam’, ‘learning_rate’: ‘adaptive’, ‘hidden_layer_sizes’: (100,), ‘beta_2’: 0.5, ‘beta_1’: 0.5, ‘alpha’: 0.0001, ‘activation’: ‘logistic’}

**Table 3:** Mean accuracy ± standard deviation across the k-folds, with *k* = 2, 4, 5, 10.

	*k* = 2	*k* = 4	*k* = 5	*k* = 10
**LSVM**	0.92 ± 0.018	0.991 ± 0.015	0.999 ± 0.002	0.999 ± 0.002
pagg	0.893 ± 0.018	0.951 ± 0.037	0.96 ± 0.026	0.982 ± 0.03
**LR**	0.902 ± 0.0	0.964 ± 0.018	0.978 ± 0.02	0.991 ± 0.018
XGB	0.556 ± 0.011	0.604 ± 0.021	0.591 ± 0.041	0.609 ± 0.119
GNB	0.644 ± 0.025	0.618 ± 0.079	0.613 ± 0.08	0.684 ± 0.133
RBF-SVM	0.511 ± 0.038	0.529 ± 0.021	0.573 ± 0.022	0.582 ± 0.076
RF	0.609 ± 0.02	0.591 ± 0.04	0.591 ± 0.05	0.596 ± 0.054
NN	0.871 ± 0.004	0.969 ± 0.019	0.973 ± 0.026	0.964 ± 0.034

**Table 4:** Calculated area under the curve for each classifier across the k-folds, with *k* = 2, 4, 5, 10.

	*k* = 2	*k* = 4	*k* = 5	*k* = 10
**LSVM**	0.919	0.991	0.999	0.999
pagg	0.891	0.948	0.959	0.982
**LR**	0.9	0.962	0.977	0.991
XGB	0.543	0.593	0.583	0.606
GNB	0.644	0.618	0.608	0.683
RBF-SVM	0.509	0.529	0.565	0.575
RF	0.571	0.549	0.548	0.552
NN	0.873	0.969	0.975	0.963

**Table 5:** Top 12 WM brain area pairs which feature correlations were highly ranked through RFE-CV selection. L or R at the end stands for the left or right hemispheres, respectively.

Retrolenticular Part of Internal Capsule L	&	Fornix Cres/Stria Terminalis
Anterior Limb of Internal Capsule L	&	Uncinate Fasciculus R
Body of Corpus Callosum	&	Tapetum L
Corticospinal Tract R	&	Posterior Corona Radiata R
Posterior Limb of Internal Capsule R	&	Retrolenticular Part Of Internal Capsule R
External Capsule R	&	Tapetum L
Middle Cerebellar Peduncle	&	Inferior Cerebellar Peduncle R
Anterior Limb of Internal Capsule R	&	Tapetum R
Middle Cerebellar Peduncle	&	Cingulum Cingulate Gyrus L
Anterior Limb of Internal Capsule R	&	Fornix Cres /StriaTerminalis R
Inferior Cerebellar Peduncle R	&	Retrolenticular Part Of Internal Capsule R
Cingulum Hippocampus L	&	Superior Fronto-occipital Fasciculus R

## Data Availability

This work uses DTI data from the ABIDE-II dataset, publicly available at http://fcon_1000.projects.nitrc.org/indi/abide/abide_II.html (accessed on 10 November 2021). DTI data were used from the downloaded sites of BNI, NYU1, NYU2, SDSU, and TCD. The website also contains phenotypic files for each site, as well as the DTI scanning parameters.

## Supplementary Materials

The following are available at https://www.mdpi.com/article/10.3390/s21248171/s1, Table S1: contains accuracy results for F representation, for different RFE-CV kernels. Table S2: contains accuracy results for $\hat{F}$ representation, for different RFE-CV kernels. Table S3: subjects’ demographics: subject IDs, along with age, label, IQ, and gender, and also the summary of those demographics for each group.

## Abbreviations

The following abbreviations are used in this manuscript:

DTI	Diffusion tensor imaging
MRI	Magnetic resonance imaging
ASD	Autism spectrum disorder
ADOS	Autism Diagnostic Observation Schedule
ADI-R	Autism Diagnostic Interview-Revised
ABIDE	Autism Brain Imaging Data Exchange
